# Increases in Awareness and Uptake of Dating Apps’ Sexual Health Features Among US Men Who Have Sex with Men, 2018 to 2021

**DOI:** 10.1007/s10461-024-04349-4

**Published:** 2024-05-09

**Authors:** Jennifer Hecht, Maria Zlotorzynska, Dan Wohlfeiler, Travis H. Sanchez

**Affiliations:** 1 Building Healthy Online Communities and Springboard HealthLab, 5601 Van Fleet Ave, Richmond, CA 94804 USA; 2 Independent Researcher, Atlanta, USA; 3 Rollins School of Public Health, Emory University, 1518 Clifton Road, Atlanta, GA 30322 USA

**Keywords:** MSM, HIV, Sexual health, Internet, Dating apps

## Abstract

Dating apps are now used by the majority of MSM to meet sexual and romantic partners. While research has demonstrated an association between app use and greater number of sex partners and STIs, dating apps also pose an opportunity for intervention. By advocating for new and improved sexual health features on dating apps, Building Healthy Online Communities (BHOC) aims to increase communication about sexual health on the apps. As a follow-up to our previous paper assessing the uptake of sexual health-related profile options on dating apps through Emory’s annual survey of 10,000 MSM in the US, BHOC and Emory partnered to explore the change in uptake over time, again through their annual survey. Among survey participants in 2021, 85% reported using dating apps to meet a partner in the past year, and among this group, 93% reported awareness of sexual health features, up from 77% in 2018 (p < 0.0001). 71% of app users who were aware of features in 2021 reported using one or more sexual health feature, up from 61% in 2018 (p < 0.0001). BHOC will continue to advocate for increased uptake of these features, especially among subgroups with lower levels of uptake.

## Background

Dating apps are now used by the vast majority of gay, bisexual and other men who have sex with men (MSM) to meet sexual and romantic partners [1–3]. Dating apps provide challenges for HIV and STI prevention efforts, since they make it easy to find new partners, and unlike bars or clubs, are often accessible for free. They also can expand sexual networks since they make it possible to quickly find new partners. They can do this both in larger cities, which already offer a number of ways to meet partners, as well as in places where apps may be the only way to find them [4]. Furthermore, numerous studies have found that MSM who meet partners online are more likely to have higher-risk sex acts, more male partners, more condomless sex, and higher rates of sexually transmitted infections [5–7]. However, dating apps can also provide opportunities for prevention. Previous studies indicate a correlation between app use and increased uptake of sexual health resources, including HIV testing and use of pre-exposure prophylaxis (PrEP). [5, 8]

Dating apps also provide opportunities to prevent HIV and STI transmission. Interventions on a dating app can reach millions of users [9], thereby supporting sexual health at a population level in contrast with other local and in-person interventions that are challenged with reaching this same scale [10]. Structural interventions on dating apps involve building features like fields in the personal profiles that users fill out to exchange important information with other users about their preferred sexual health strategies (i.e., condom use, taking PrEP, and maintaining an undetectable viral load) as well as HIV status and testing history [9]. This can often be easier than exchanging sexual health information in person [11]. By exchanging sexual health information, users can make more informed decisions about prospective partners. Many apps also include links to important sexual health information and resources, such as online HIV/STI testing, PrEP, and care locators.

Many of these features were created with the encouragement and support of Building Healthy Online Communities (BHOC), a consortium of public health leaders and gay dating website and app owners working together to support HIV and STI prevention online (https://bhocpartners.org). BHOC’s intention in doing so was to help dating apps implement structural interventions, which provide a sustainable benefit with a broad reach without relying on the scarce financial and staff resources of public health programs. Since 2017, BHOC has partnered with Emory University’s American Men’s Internet Survey (AMIS) to assess trends in MSM utilization of dating apps, which sexual health features they utilize, and other potential features users may support. We have previously reported 2018 data on the percentage of dating app users who were aware of sexual health features on dating apps and the prevalence of their use [9]. Disruptions due to COVID-19 clearly impacted many behaviors of MSM, including changing their patterns of dating app usage [12]. This study examines changes in awareness and utilization of dating app sexual health features from nationwide samples of the annual AMIS surveys conducted in 2018 and 2021. We also examine uptake of these features among those who report using PrEP as a prevention strategy.

## Methods

Annual cross-sectional American Men’s Internet Survey (AMIS) aims to collect 10,000 complete surveys from eligible cisgender MSM 15 + years of age who reside in the US. Detailed recruitment, enrollment, and data methods have been previously reported [13, 14]. Briefly, participants were recruited through convenience sampling using banner ads placed on a variety of websites and apps, including general social networking sites and dating apps. Men who clicked on ads were directed to the survey website hosted on a secure server administered by Alchemer (Boulder, CO), where they answered a short screener to determine eligibility. Eligibility criteria included age 15 years or older, male sex at birth and male gender identity, residence in the United States, and oral and/or anal sex with a male partner at least once in the past. Eligible participants were shown an electronic informed consent form (or assent form, for those ages 15 to 17) and asked to affirm consent or assent. Those who consented continued on to complete the survey.

The study was conducted in compliance with federal regulations governing protection of human subjects and was reviewed and approved by Emory University’s Institutional Review Board. No incentive was provided to the participants.

The dataset of eligible responses underwent several data cleaning steps, as described previously. First, survey responses were deduplicated by internet protocol (IP) address, retaining the observation with the highest survey completion. Then the dataset was limited to those observations with no missing values for the first question of at least two consecutive sections. Finally, the analytic dataset included only those participants who reported having oral or anal sex in the past 12 months and who provided a valid US zip code.

Awareness was assessed with a question about whether dating app users knew that they could list their preferred sexual health strategy (such as condoms, PrEP, and being on treatment for HIV) on the app, and for those who indicated awareness, utilization was assessed by asking whether dating app users had utilized these features and which ones they opted in to.

This analysis included only those who reported using a dating app within the prior 12 months. Participant demographic characteristics included age, race/ethnicity, self-reported HIV status, primary language and county of residence population density [15]. Behavioral characteristics were assessed for the 12 months preceding the survey and included condomless anal intercourse with a male partner, number of male sexual partners and partner type. Partner type was classified as only main partners, only casual partners or both main and casual partners.

Poisson models using Generalized Estimating Equations (GEE) were used to test for a linear trend between AMIS-2018 and AMIS-2021 for each of the outcomes (awareness and uptake of profile options) stratified by self-reported HIV-status (positive and negative/unknown). Trends in awareness and usage were also assessed for sub-groups of MSM reporting specific sexual behaviors in the past 12 months: condomless anal sex and having two or more sexual partners. Trends in usage of specific profile options were assessed for participants who reported use of any profile options: condoms, being on PrEP (among HIV-negative or unknown status participants who used PrEP in the past 12 months), being on treatment for HIV (among HIV-positive participants who were currently taking antiretroviral medication). All models controlled for age (continuous), race/ethnicity, HIV status (if applicable), urban/rural residence, primary language, condomless anal sex with a male partner, number of male sex partners, and male partner type in the past 12 months.

A similar Poisson model using GEE was used to calculate an adjusted prevalence ratio (aPR) and 95% confidence interval (CI) for the association between usage of profile options for those who reported using PrEP in the past 12 months vs. those who did not, among HIV-negative and unknown status participants, adjusting for age (continuous), race/ethnicity, urban/rural residence, primary language, condomless anal sex with a male partner, number of male sex partners, and male partner type in the past 12 months.

## Results

In 2018, of the 10,129 MSM who answered the AMIS-2018 survey, 6737 (66.5%) reported using dating websites or apps to meet other men in the past 12 months and 6525 (64.4%) completed the questions specific to dating app usage. In 2021, 7691 MSM (84.9%) reported using a dating app in the past 12 months and answered the additional questions relating to dating app usage (n = 6202, 80.6%). Demographic and behavioral characteristics of 2021 participants are shown in Table 1. Over half of the sample was older than 40 and approximately two-thirds described their race or ethnicity as non-Hispanic white. The prevalence of self-reported HIV seropositivity was 15.7% (976/6202). Over three-quarters of the sample reported having condomless anal sex in the past 12 months and 87.1% (5399/6202) had two or more male partners in that time period.

**Table 1:** Demographic and sexual behavior characteristics of US MSM who used dating websites or apps to meet men in the past 12 months, American Men’s Internet Survey, 2021

Characteristic	N
Total	6202
Age	
15–17	11 (0.2)
18–24	399 (6.4)
25–29	572 (9.2)
30–39	1451 (23.4)
40 and older	3769 (60.8)
Race/ethnicity	
Black, non-Hispanic	801 (12.9)
Hispanic	707 (11.4)
White, non-Hispanic	4156 (67.0)
Other or multiple races	459 (7.4)
County population density	
Urban	2949 (47.5)
Suburban	1299 (20.9)
Small/medium metro	1512 (24.4)
Rural	430 (6.9)
Primary language	
English	5924 (95.5)
Spanish	211 (3.4)
Other	59 (1.0)
HIV status	
Positive	976 (15.7)
Negative/unknown	5226 (84.3)
Condomless anal sex, past 12 months	
Yes	4901 (79.0)
No	1301 (21.0)
Sex partners, past 12 months	
One	584 (9.4)
Two or more	5399 (87.1)
Partner type(s), past 12 months	
Main only	603 (9.7)
Casual only	2542 (41.0)
Main and casual	2795 (45.1)

**Fig. 1:**
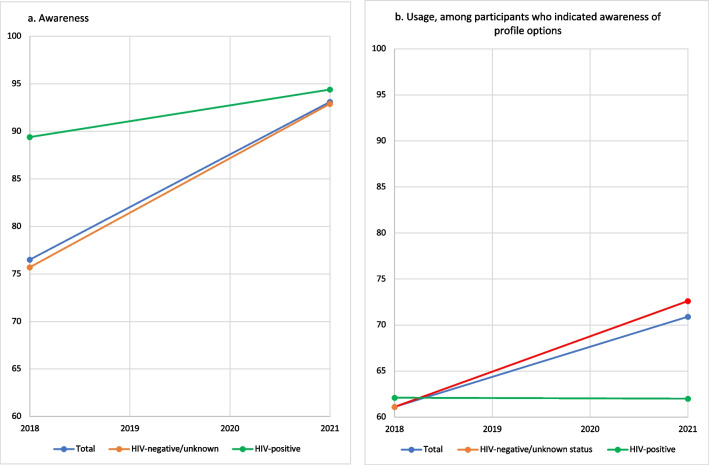
Proportion of US MSM participants reporting (**a**) awareness; and (**b**) usage of dating application profile options among MSM who used dating websites or apps to meet men in the past 12 months, by HIV status and year, American Men’s Internet Survey, 2018–2021

We found significant differences in awareness and uptake of dating app features between 2018 and 2021 (Fig. 1). Between 2018 and 2021 awareness of dating app sexual health features significantly increased (χ^2^ = 330.84, p < 0.0001) among all participants from 76.5% (4993/6525) in 2018 to 93.1% (5777/6202) in 2021 (Fig. 1a). Between 2018 and 2021 use of dating app sexual health features significantly increased (χ^2^ = 73.18, p < 0.0001) among all participants from 61.1% (2886/4721) in 2018 to 70.9% (3921/5528) in 2021 (Fig. 1b).

Among MSM who were HIV-negative or had an unknown status, the percentage of participants reporting awareness increased from 75.7% (4623/6111) in 2018 to 92.9% (4856/5226) in 2021 (χ^2^ = 302.73, p < 0.0001) (Fig. 1a), while usage increased from 61.1% (2665/4365) in 2018 to 72.6% (3372/4643) in 2021 (χ^2^ = 80.76, p < 0.0001) (Fig. 1b). Among HIV-positive users, awareness increased from 89.4% (370/414) in 2018 to 94.4% (921/976) in 2021 (χ^2^ = 12.79, p = 0.0003) (Fig. 1a). There was no significant change in usage among HIV-positive men (62.1%, 221/356, to 62.0%, 549/885) from 2018 to 2021 (Fig. 1b).

**Fig. 2:**
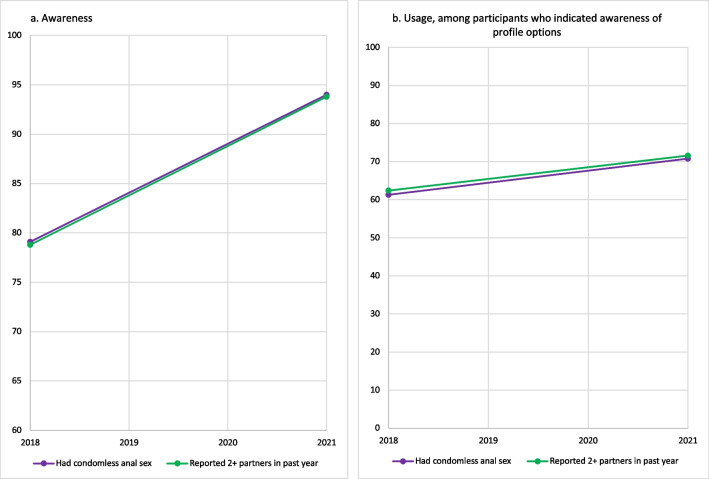
Proportion of US MSM participants reporting (**a**) awareness; and (**b**) usage of dating application profile options among MSM who used dating websites or apps to meet men in the past 12 months, by past-year sexual behavior and year, American Men’s Internet Survey, 2018–2021

Among men who reported having condomless anal sex in the previous 12 months, awareness increased from 79.1% (3688/4661) to 94% (4608/4901) (χ^2^ = 266.98, p < 0.0001, Fig. 2a) and usage increased from 61.3% (2145/3501) in 2018 to 70.8% (3136/4427) in 2021 (χ^2^ = 52.39, p < 0.0001, Fig. 2b). Among men who had two or more partners in 2018, 78.8% (χ^2^ = 296.67, 4318/5482) were aware of profile options (Fig. 2a); in 2021, this increased to 93.8% (5062/5399, p < 0.0001). In this group, usage increased from 62.4% (2550/4088) to 71.6% (3489/4873) (χ^2^ = 62.77, p < 0.0001, Fig. 2b).

Within the three different sexual health strategies, we saw increases in all (Table 2): 58.4% (2288/3921) reported opting into the condom use profile option in 2021 compared to 49.5% (1430/2886) in 2018 (χ^2^ = 83.19, p < 0.0001), 97.4% (1755/1802) of those who took PrEP in the past 12 months reported opting into the PrEP profile field compared to 64.2% (498/776) in 2018 (χ^2^ = 267.95, p < 0.0001), and among those on ART, 89.2% (477/535) reported opting into the profile field that indicated treatment as prevention compared to 63.7% in 2018 (130/204, χ^2^ = 43.16, p < 0.0001).

**Table 2:** Use of specific dating profile options by US MSM who used dating websites or apps to meet men in the past 12 months and were aware of profile options, by year, American Men’s Internet Survey, 2018–2021

Profile options used	2018			2021			Chi-square^a^	p-value^b^
	N	n	%	N	n	%		
Condoms	2886	1430	49.5	3921	2288	58.4	83.19	< 0.0001
PrEP	776^c^	498	64.2	1802^b^	1755	97.4	267.95	< 0.0001
Treatment as prevention/taking HIV meds/undetectable	204^d^	130	63.7	535^c^	477	89.2	43.16	< 0.0001

^a^Chi-square value for AMIS cycle variable, based on Generalized Estimating Equation model of linear test for trend that controlled for age (continuous), race/ethnicity, urban/rural residence, primary language, condomless anal sex with a male partner, number of male sex partners and male partner type in the past 12 months

^b^Chi-square p-value for AMIS cycle variable, based on Generalized Estimating Equation model of linear test for trend that controlled for age (continuous), race/ethnicity, urban/rural residence, primary language, condomless anal sex with a male partner, number of male sex partners and male partner type in the past 12 months

^c^Includes HIV-negative/unknown status participants who used PrEP in the past 12 months

^d^Includes HIV-positive participants who were currently taking antiretroviral medication

Among 2021 participants aware of the features and who reported HIV-negative or unknown status, we found higher rates of utilization among those who reported using PrEP in the past 12 months than those who did not (85.7%, 1802/2102 vs. 61.1%, 1485/2430, aPR = 1.38, 95% CI 1.33, 1.43).

## Discussion

We note that our efforts to increase access to and uptake of sexual health features on gay dating apps is supported by research suggesting that Grindr users have higher rates of HIV risk but also are more open to PrEP [5].There have been significant and substantial increases in US MSM’s awareness of dating app sexual health features since we last reported on this data [9] and we may be close to reaching saturation in this awareness. There are also significant and substantial increases in utilization of these features, but opportunities still remain to increase uptake, particularly among some groups of MSM, like those who are HIV positive. We saw a very small increase in awareness among HIV + users, but no significant increase in utilization among this group. Given the noted association between dating app use and vulnerability of acquiring HIV or STIs, further efforts to understand barriers to uptake are warranted [6]. Exploring whether this overall lower uptake and the lack of increase over time is related to HIV stigma, or other factors, would be a useful next step to ensure broad engagement in sexual health features. Notably, PrEP uptake is also lower among adolescents [16]. While there were some differences in awareness and uptake across a number of demographic and behavioral factors, we note these were relatively small differences, with the exception of the youngest participants; participants who were 15–17 years old were only 67% as likely to report awareness of features and 40% as likely to use them compared to the reference group of 40 years or older. While we note a very small sample size of less than 20 in this age group, and according to the terms of service of the dating apps these participants were too young to legally be on the apps, we recognize the opportunity to increase awareness and uptake of these features among youth.

We note several limitations to the present study. First, these data were collected through an online convenience sampling approach and may not be generalizable to all MSM in the US or all MSM online. Second, in some cases our terminology may not have been clear to study participants. For example, the term “sexual health strategies” may not have been a term that all participants understood. Third, while participant language was not found to be significantly associated with knowledge of profile options, our sample was likely biased as the AMIS survey was administered only in English during the 2018 survey; Spanish was added for 2021. Finally, not all apps have the same profile options available to users. For example, Grindr, the most commonly-used dating app, does not have an option to indicate condom use, although users can indicate their HIV status and/or PrEP use/being undetectable. While most of the apps include sexual health options as part of users’ profiles, apps differ in which options are included, and the wording of each option. We note that, as with many communications—both online and offline–there is no process for verification, and some users will choose to lie or leave out important health information. We also acknowledge the data collection occurring during the COVID pandemic may have affected participants’ use of dating apps.

## Conclusions

While using dating apps to find new partners is correlated with risk, dating apps also provide many opportunities to support MSM sexual health. Apps can reach millions of users with information about outbreaks, prevention strategies, and provide links to health information. However, this requires ongoing investments by public health. On the other hand, nearly all apps have built-in features which allow users to disclose and find others’ prevention strategies, thus allowing them to make informed decisions about their partners. This is particularly useful for those who find it difficult to have these conversations in person. These features are permanent, and provide a valuable prevention strategy without requiring any cost to public health. Dating apps can also remind users of the need to get tested through built-in features.

We are encouraged by the increase in awareness and utilizations of these features. Nevertheless, more efforts are needed in order to increase both awareness and utilization. Dating apps should periodically encourage users to utilize these features, and educate them about the pros and cons of disclosing sexual health information online. HIV and STI prevention staff and healthcare providers should do so as well.

## Data Availability

Not applicable.
